# Education in wound care: Curricula for doctors and nurses, and experiences from the German wound healing society ICW

**DOI:** 10.1186/s40779-016-0094-1

**Published:** 2016-09-06

**Authors:** Karl-Christian Münter

**Affiliations:** Academic Practice Affiliated to University of Hamburg, 22177 Hamburg, Germany

**Keywords:** Chronic wounds, Wound healing, Treatment

## Abstract

The German wound healing society ICW (Initiative Chronische Wunden) started a training program for nurses in 2005. Certified by TÜV Rheinland the courses are regularly audited and the quality is ensured. More than 30000 nurses attended these courses in Germany. In three Chinese hospitals the ICW courses have been adopted. A close collaboration between Chinese and German experts in wound healing let to their further development and improvement. The article reviews the need for education in the treatment of chronic wounds for medical personnel - nurses and doctors. Experiences and perspectives are discussed.

## Introduction

**Fig. 1:**
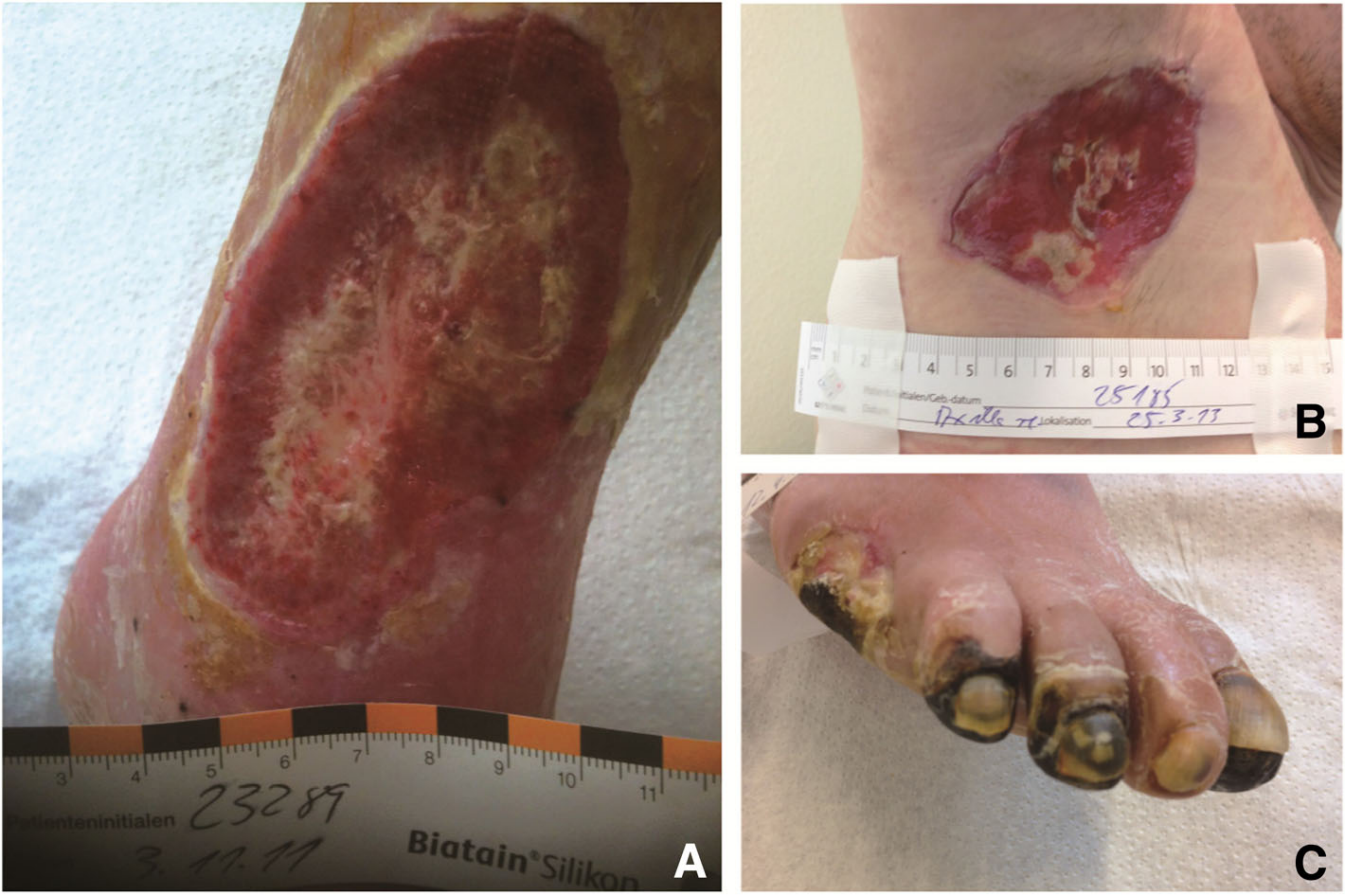
Chronic wounds. **a** Ulcer in chronic venous disease; **b** Hidradenitis suppurativa (acne inversa); **c** Peripheral arterial disease

The topic of chronic wounds has become increasingly important in all developed countries of the world. Demographics clearly show that the population is aging. In 2011 in China, 15% of the population was above the age of 60. In 2030, it is estimated that this percentage will have reached 30%. Standards of living have risen in recent years, and the health system has improved. On the other hand, studies have shown that many people have gained weight and moved less. In consequence, non-infectious chronic diseases, such as hypertension, diabetes mellitus, and hyperlipidemia, are on the rise. In the course of these diseases, chronic wounds may develop, i.e., wounds lasting more than eight weeks, and with an underlying cause (Fig. 1).

The treatment of chronic wounds can be frustrating and expensive. In Europe, 25% of all diabetes mellitus related costs for hospital care are due to the treatment of the diabetic foot syndrome (DFS) [1, 2]. Chronic wounds have become a significant problem for health care systems. In Germany, between 0.08% and 1.0% of the population suffer from venous leg ulcers (around 800 000 patients) [3]. In recent studies, based on secondary data from statutory health insurances, the costs per treatment of a venous ulcer are 7 631 Euro [4]. In a multicentre study the treatment costs for diabetic foot ulcerations which healed were 7 722 Euro. Patients who died before healing created costs of 8 953 Euro and those who underwent major amputations needed 25 222 Euro [5]. The average costs for the diabetic foot syndrome arise to 15 000 Euro per year [6]. Evidently, only a systematic approach to treatment could be successful while cutting costs.

Unfortunately, the principles for the treatment of chronic wounds are not included in the curricula of doctors and nurses worldwide, which leaves health care professionals largely to their own educational development, and patients experience disparate treatment quality, depending on the individual quality of the centers to which they are admitted.

The German wound healing society Initiative Chronische Wunden (ICW) initiated structured courses for health care professionals in 2005. In Germany, more than 30000 nurses have successfully participated in recent years. So far, these courses have been used in three Chinese hospitals as well. Experiences and perspectives will be discussed in this article.

### The ICW

The ICW is a German wound healing society with more than 4000 members. Its purpose is the promotion of modern wound healing. From its inception, the ICW included doctors and nurses in its membership as well as in its governances, such as the board (6 members) and the council (approximately 20 members) in order to underline the special necessity of teamwork in the treatment of chronic wounds. The rationale for founding the society was the lack of optimal treatments for patients with chronic wounds in Germany despite the existing knowledge and resources. In 2002, the ICW adopted the status of “eingetragener Verein “(“e.v.”) stating that the ICW is a nonprofit organization; all revenues have to be reinvested in projects related to wound healing.

### The “wound expert“

Soon, it became clear that isolated congresses and lectures would not change the actual daily practice in the hospitals and the community. A more systematic approach was needed. In 2005, the first courses, called “Wundexperte“ or ‘wound expert’, could be offered. The curricula of these courses were developed by a group of experts, some of which included teachers of nursing who were ICW members. The courses included coverage of anatomy, pathophysiology and therapy of chronic wounds. A special effort was made to include the perspectives of the patient, the role of the families and other caretakers, and the importance of wound–related topics, such as nutrition and footwear. A textbook addressing these subjects has been published, has already been revised, and can be used as a resource during the courses. All legal rights of the materials are held by the ICW, but the courses are not offered by the ICW itself. The organization of a specific course lies with its distributors, who may be, for example, nursing schools or home care enterprises. As the courses are premiered throughout Germany, the question arose regarding how quality could be ensured among a divergent crowd of distributors. The Technischer Überwachungsverein (TÜV) Rheinland is a government-approved organization, which controls the quality in various branches, most often in technical projects, such as those involving cars. The ICW approached the TÜV and asked for indefinite control of its content, as well as the organization of all courses held in Germany on behalf of the ICW, including the qualification of lecturers. Then, a partnership between ICW and TÜV Rheinland was established. Courses will now be audited on an unpredictable basis and without any warning. Distributors who do not comply with the regulations will be barred from further courses. As a means of ensuring its own quality control, ICW also accepts oversight and regular recertification by the TÜV.

These measures secure an ongoing high quality and actualization of the course curriculum, which is regularly updated by the curriculum’s group.

In Germany, the courses are offered to all health care professionals who offer wound healing in any aspect of their daily practice. Of course, nurses and doctors are invited; however, podiatrists and other related professionals (i.e. pharmacists) have participated throughout the years. The course comprises a one-week school term and a two-day visitation in a wound healing institution. Upon completion of the visitation portion of the course, a written examination follows, and a case report must be delivered.

As the purpose of the course is to ensure a constant qualified workforce in wound healing, participants must be recertified every five years. During these years, they have to participate regularly in ICW-certified education, such as weekend courses, or in congresses where wound healing is discussed.

The courses are offered in more than 120 institutions throughout Germany.

### Driving things further

The frequent turnover in nursing staff is one of the problems existing in Germany. Nurses may leave their jobs due to a relatively low income and high workload or for family reasons. Therefore, there is a constant need for the education of beginners in wound healing, and the basic course “wound expert“is still needed.

Nevertheless, others are eager to qualify themselves further, and the role of advanced education to an academic level in nursing becomes even more important. The ICW has taken this development into account and is now offering two more advanced courses for those who passed the basic course and have proven that they are continuing to the field of wound healing. These courses are open to nurses, but not to all other health care professionals because these courses will qualify their participants for higher positions in the nursing staff of hospitals and related institutions (e.g., nursing homes.) One course is focused on those who want to learn more about the medical background of wound healing and who wish to acquire advanced techniques, such as local debridement. The aim is to qualify participants for a leading position in a wound ambulance or wound healing unit in a hospital. The other course is more directed towards those who want to pursue a career in hospital administration. Organization of wound care in a cost-effective manner is becoming paramount, and advanced management skills are therefore needed.

### Constant medical education – the role of doctors

As mentioned above, the basic course, “Wundexperte“, is open to all health care professionals, including nurses, practitioners of any kind, and doctors. Compared to other groups, very few doctors have taken part in these courses thus far. A certain reluctance has dominated the approach of doctors to chronic wounds in the past, leaving the actual wound care in the hands of the nurses, without acknowledging that doctors play a crucial role in the therapeutic team. This is only slowly changing.

To promote further engagement of doctors, the ICW is now offering a course exclusively for this group. Pathologic and anatomic background as well as advanced techniques of diagnosis and therapies are the contents of this course. Participants must be graduates who have also finished specialization of any type. During the initial offerings of the course, interest has been raised by general practitioners, geriatricians, internists, and dermatologists, as well as general, plastic and vascular surgeons.

### ICW in China

Through the initiative of the German company Hartmann (producer of wound care products), associations have been established between three Chinese hospitals and the ICW. The basic course, “Wundexperte“, has been held several times, and approximately 300 Chinese nurses passed the exam to date. As the course concludes with the official ICW certification, no compromises are made concerning the contents. Additionally, the usual audits by ICW-approved auditors take place (as travel arrangements have to be made, the audits are planned, and the dates are known in advance).

However, there are already adaptations to the specific situation in China. For example, most of the course participants come from hospitals far away from the center where the actual course takes place. They normally stay there in dormitories for two months. Two weeks are devoted to studies, and six weeks are used to combine the newly adopted knowledge with practical work in a wound caring ward in their host hospital. Therefore, practical work is much more of a focus in China than it is in Germany, which is a remarkable improvement. The ongoing dialogue between the Chinese and German partners will lead to further improvement of the quality of the courses in both countries.

### Perspectives

The ICW is a non-profit organization and does not gain any benefits from the courses. However, financial aspects must be considered. Qualified lecturers and auditors must be compensated, and rooms, materials, and miscellaneous items may contribute to the amount of money needed to run one of the courses. So far, only one company supports ICW courses in China (Hartmann). Obviously, this can only ensure the performance quality of the courses on a very limited scale.

Considering all of the different circumstances in our two countries, some problems seem to be quite similar. The problem of dealing with the increasing number of chronic wounds and the lack of qualified treating personnel is one of them. Cooperation in which we learn from each other is therefore highly appreciated. The ICW is eager to contribute to this cooperation with existing course programs.

Possible adaptations to specific Chinese necessities are required and can be discussed in the friendly and productive atmosphere, which we have experienced in the last few years. Such a discussion may lead to improvements in the courses in unforeseen aspects.

Wound healing has become a broader and more international topic in recent years, and the formation of the Asian Wound Healing Association proves this. Expanding our perspective to a truly worldwide cooperation is an exciting adventure and a perfect possibility to pave the way to mutual understanding.

## Abbreviations

DFS: Diabetic foot syndrome
ICW: Initiative Chronische Wunden
TÜV: Technischer Überwachungsverein
