# Erratum to: Education in wound care: Curricula for doctors and nurses, and experiences from the German wound healing society ICW

**DOI:** 10.1186/s40779-016-0111-4

**Published:** 2017-02-03

**Authors:** Karl-Christian Münter

**Affiliations:** Gemeinschaftspraxis Bramfeld, Bramfelder Chaussee 200, Hamburg, 22177 Germany

## Erratum

Following publication of this article [1], it has come to our attention that the incorrect version of the manuscript was published. In this incorrect version, the title of the article has been updated to “Education in wound care: Curricula for doctors and nurses, and experiences from the German wound healing society ICW”. The author affiliations in the Frontmatter were also captured incorrectly as “^1^Gemeinschaftspraxis Bramfeld, Bramfelder Chaussee 200, Hamburg 22177, Germany. ^2^Akademische Lehr—und Forschungspraxis, der Universität Hamburg, Hamburg, Germany” however, instead should only be “^1^Gemeinschaftspraxis Bramfeld, Bramfelder Chaussee 200, Hamburg 22177, Germany”. Furthermore, the abbreviations and author’s information was captured incorrectly, and references section was missed in the article, which should have included the following:

**Abbreviations**

DFS, Diabetic foot syndrome; ICW, Initiative Chronische Wunden; TÜV, Technischer Überwachungsverein

**Competing interests**

The author declares that he has no competing interests

**Author’s information**

Karl-Christian Münter, MD, Phlebologist, Board member ICW (Initiative Chronische Wunden), Council member EWMA (European Wound Management Association), Academic Practice Affiliated to University of Hamburg, Germany.

**References**

1. Liebl A, Neiss A, Spannheimer A, Reitberger U, Wagner T, Görtz A. Costs of type 2 diabetes in Germany. Results of the CODE-2 study. Dtsch Med Wochenschr. 2001;126:585–9.

2. Meyer-Heintze V. [Diabetic foot from the social medicine viewpoint]. Zentralbl Chir. 1999;124 Suppl 1:45–8.

3. Arbeitsgruppe Erhebung und Nutzung von Sekundärdaten (AGENS) der Deutschen Gesellschaft für Sozialmedizin und Prävention: Gute Praxis Sekundärdatenanalyse (GPS). In: Swart E, Ihle P (Hrsg): Routinedaten im Gesundheitswesen. Handbuch Sekundärdatenanalyse: Grundlagen, Methoden, Perspektiven. Bern: Verlag Hans Huber 2005; 405-12. [Publication in German].

4. Quan H, Sundararajan V, Halfon P, Fong A, Burnand B, Luthi JC, et al. Coding algorithms for defining comorbidities in ICD-9-CM and ICD-10 administrative data. Med Care. 2005;43:1130–9.

5. Prompers L, Huijberts M, Schaper N, Apelqvist J, Bakker K, Edmonds M, et al. Resource utilisation and costs associated with the treatment of diabetic foot ulcers. Prospective data from the Eurodiale Study. Diabetologia. 2008;51:1826–34.

6. Girod I, Valensi P, Laforêt C, Moreau-Defarges T, Guillon P, Baron F. An economic evaluation of the cost of diabetic foot ulcers: results of a retrospective study on 239 patients. Diabetes Metab. 2003;29:269–77.
